# Cost-effectiveness analysis of a prediction model for community-based screening of active tuberculosis

**DOI:** 10.7189/jogh.14.04226

**Published:** 2024-11-22

**Authors:** Chih-Chi Yang, Yun-Ju Shih, Helen Ayles, Peter Godfrey-Faussett, Mareli Claassens, Hsien-Ho Lin

**Affiliations:** 1 Institute of Epidemiology and Preventive Medicine, National Taiwan University, Taipei, Taiwan; 2 Clinical Research Department, Faculty of Infectious and Tropical Diseases, London School of Hygiene & Tropical Medicine, London, UK; 3 Zambart, University of Zambia School of Public Health, Ridgeway, Zambia; 4 Desmond Tutu TB Centre, Department of Paediatrics and Child Health, Faculty of Medicine and Health Sciences, Stellenbosch University, Cape Town, South Africa; 5 Department of Human, Biological and Translational Medical Sciences, School of Medicine, University of Namibia, Windhoek, Namibia

## Abstract

**Background:**

Active case finding could effectively detect tuberculosis (TB) patients, but it can be costly. Therefore, a feasible, cost-saving, and efficient algorithm for community-based TB screening is needed.

**Methods:**

The study population was based on a previous TB prevalence survey conducted in the Zambia/South Africa Tuberculosis and HIV/AIDS Reduction trial. We developed predictive scoring models for HIV-positive and HIV-negative/unknown populations for practical purposes. We compared the cost-effectiveness of our models with that of WHO tools through average cost-effectiveness ratio (ACER) and incremental cost-effectiveness ratio.

**Results:**

The prediction model for HIV-positive population presented higher area under the curve (AUC) = 0.652, 95% confidence interval (CI) = 0.602–0.701) than WHO-recommended four-symptom screen (AUC = 0.568, 95% CI = 0.524–0.612) among South African participants. The AUC of the model for HIV-negative/unknown population was 0.673 (95% CI = 0.648–0.697), which was higher than that of the WHO tools as well. The ACER of our model can range from 246 to 1670 USD per TB case detected in the South African communities.

**Conclusions:**

The scoring system for active TB case finding presented a better performance and showed cost-effectiveness, which can provide new strategies for active TB case finding with multiple options under budget considerations.

Undetected tuberculosis (TB) cases are a challenge to global TB control [1], emphasised in the End TB Strategy implemented by the World Health Organization (WHO) [2]. These cases could contribute to pathogen transmission, morbidity and mortality, due to the lack of access to health care facilities for prompt diagnosis and treatment, especially in high burden low resource settings. Active case finding (ACF) could decrease the transmission of TB by providing early-phase treatment and fundamentally altering the epidemiology of TB at the community level [3]. Nevertheless, performing ACF is costly, requiring human resources, time, and equipment such as screening tools and diagnostic tools [4].

According to the systematic screening principles proposed by WHO [5], when deciding on a particular algorithm, the accuracy, accessibility, feasibility and cost of screening strategies and diagnostic tools should be considered and evaluated. In the latest guideline [6], WHO still recommended symptom-based algorithms for screening, such as coughing more than two weeks (termed ‘prolonged cough’), any presence of cough, fever, weight loss and night sweats (termed ‘WHO-recommended four-symptom screen (W4SS)), and W4SS with haemoptysis (termed ‘any TB symptom’). The WHO-recommended four-symptom screen was widely used in resource-constrained countries among the HIV-positive population, while the other two were used for screening among the general population [6].

However, these symptom-based algorithms had an unstable accuracy to detect active TB [6–8], which might result in the detection of fewer TB cases and many non-TB individuals needing further diagnostic tests, causing a heavy burden for resource-limited countries. Furthermore, the lack of specificity of the W4SS rule challenged the capacity of TB diagnostic laboratories [6,9], indicating the need for an alternative screening algorithm for TB.

Our study follows the latest WHO guidelines and address the deficiency in cost-effectiveness research. We developed and validated a prediction model for active TB case finding in a community setting according to the suggestion from a systematic review [10]. The prediction model was then converted to a pragmatic scoring system. Second, we compared our scoring system with the symptom-based tools proposed by WHO by conducting cost-effectiveness analyses. Through the evaluation of cost and yield, we aimed to determine critical strategies with both efficiency and cost-saving for active TB case finding among competing algorithms.

## METHODS

The study flow is shown in Figure S1 in the **Online Supplementary Document**. Model development and validation was followed by cost-effectiveness analyses of the case finding strategies [11].

### Study population

The study population was obtained from a prevalence survey conducted in 2010 as part of the Zambia/South Africa Tuberculosis and HIV/AIDS Reduction (ZAMSTAR) trial [12]. Data were collected from eight communities in South Africa and 16 communities in Zambia. We used the South African population for the development of prediction model and the Zambian population for external model validation (Figure S2 in the **Online Supplementary Document**). Individuals who received TB treatment at the time of data collection were excluded because treatment might affect presenting symptoms. We developed separate prediction models for HIV-positive and HIV-negative/unknown populations, since TB symptoms and signs of participants with HIV infection may be different from those who are HIV-negative/unknown [13]. The ethics approval of the ZAMSTAR trial has been described elsewhere [12]. An additional ethics approval for the current study was obtained from Stellenbosch University (S14/09/178).

### Measurement of outcome

Prevalent culture-confirmed TB was the outcome used in the prediction model. All participants, independent of symptoms, were asked to provide sputum specimens and defined as a case when *Mycobacterium tuberculosis (Mtb)* was detected in the sputum. Mycobacteria Growth Indicator Tube (Becton, Dickinson and Company, Franklin Lakes, New Jersey, US) liquid culture was used as the gold standard TB diagnostic test [14,15]. Laboratory methods on *Mtb* detection have been described elsewhere [16].

### Measurement of predictors

Potential predictors could be categorised into three groups: TB-related symptoms, TB risk factors and previous TB history. TB-related symptoms included shortness of breath, productive cough, current cough, chronic cough, haemoptysis, chest pain, fever, night sweats and weight loss. TB risk factors included sex, age, a history of ever drinking alcohol (‘ever drink’), a history of ever smoking cigarettes (‘ever smoke’), body mass index (BMI), and diabetes mellitus (DM) history [17,18]. Household TB history was also included and determined by the status of any member living in the same household who has ever had TB disease. Personal TB history and household TB history were mutually exclusive. All information was collected by self-report through a structured questionnaire, by trained research assistants working in the communities.

### Statistical analysis of the prediction model

The prediction model was developed through the construction of a multivariable logistic regression model; predictors were selected by the process of stepwise backward elimination based on the Akaike information criterion (AIC) [19]. Each predictor in the final model corresponded to a score, which was derived from dividing the beta coefficient by the smallest coefficient in the model and then rounding to the nearest integer [20]. Receiver operating characteristic (ROC) curves were plotted and the area under the curve (AUC) was calculated to evaluate the performance of the scoring system and WHO tools with 95% confidence intervals (95% CIs). Internal validation was done by replicating the process of model selection, score transformation and AUC computation on 1000 bootstrapped samples. AUC was used as an indicator for the evaluation of the process of model development. As a process of external validation, the final selected model was tested on a separate data set with the same data-collection method as the training data set. Analyses were performed in *R* version 3.6.3 (R Core Team, Vienna, Austria, 2020).

### Cost-effectiveness analysis (CEA) of screening algorithms

Table S1 in the **Online Supplementary Document** shows the possible combinations of screening strategies for HIV-positive and HIV-negative/unknown populations, including the scoring systems and the symptom-based algorithms recommended by WHO. Figure S3 in the **Online Supplementary Document** shows the flow of TB case finding from screening to further diagnostic tests. In the cost-effectiveness analysis, cost estimates only included costs of diagnostic tools following the screening results, and the effectiveness was defined as the number of TB cases that could be detected through the specific screening strategy and the diagnostic algorithm. The cost, sensitivity and specificity of the confirmatory tools in the algorithms are shown in Table S2 in the **Online Supplementary Document**. The average cost-effectiveness ratio (ACER) and incremental cost-effectiveness ratio (ICER) were calculated to measure the cost-effectiveness of screening strategies among the competing algorithms. Furthermore, hypothetical scenarios and the results of a community-cluster-randomised trial conducted in Zimbabwe were applied to test the scoring system as an extended application [3]. Text S1 in the **Online Supplementary Document** describes the process of CEA in detail.

## RESULTS

### Characteristic of study participants

**Table 1** and **Table 2** shows the characteristics of the study population. The majority of the participants were female, with a median age of 29 years (range = 18–103). Among the participants with HIV infection, the prevalence of TB was 4.5% (95% CI = 3.8–5.3%) and 1.6% (95% CI = 1.3–2.0%) in the South African and Zambian population, respectively. Tuberculosis prevalence was 2.0% (95% CI = 1.8–2.1%) in South Africa and 0.36% (95% CI = 0.3–0.4%) in Zambia among the HIV-negative/unknown participants.

**Table 1:** Characteristics of the HIV-positive population

Variables	South Africa			Zambia		
	**TB prevalence = 4.5%**			**TB prevalence = 1.6%**		
	**TB**	**no TB**	*P*-value*	**TB**	**no TB**	*P*-value*
	**(n = 129)**	**(n = 2714)**		**(n = 79)**	**(n = 4763)**	
Sex			0.020			<0.001
*Male*	39 (30.2%)	576 (21.2%)		34 (43.0%)	1108 (23.3%)	
Age, years			0.213			0.655
*x̄ (SD)*	34.9 (10.5)	33.7 (9.75)		34.1 (9.71)	34.6 (10.3)	
*Missing*					13 (0.3%)	
BMI, kg/m^2^			<0.001			<0.001
*x̄ (SD)*	23.7 (5.17)	27.1 (6.33)		20.2 (3.03)	22.5 (4.00)	
*Missing*	42 (32.6%)	1022 (37.7%)		7 (8.9%)	450 (9.4%)	
Ever smoke			0.009			<0.001
*Yes*	36 (27.9%)	497 (18.3%)		23 (29.1%)	668 (14.0%)	
Ever drink			<0.001			0.003
*Yes*	82 (63.6%)	1270 (46.8%)		58 (73.4%)	2656 (55.8%)	
Productive cough			<0.001			<0.001
*Yes*	42 (32.6%)	425 (15.7%)		38 (48.1%)	574 (12.1%)	
Cough			<0.001			<0.001
*Yes*	47 (36.4%)	494 (18.2%)		46 (58.2%)	809 (17.0%)	
Fever			0.297			<0.001
*Yes*	35 (27.1%)	618 (22.8%)		31 (39.2%)	404 (8.5%)	
Night sweats			0.002			<0.001
*Yes*	42 (32.6%)	562 (20.7%)		22 (27.8%)	454 (9.5%)	
Weight loss			0.001			<0.001
*Yes*	45 (34.9%)	601 (22.1%)		40 (50.6%)	928 (19.5%)	
Haemoptysis			0.014			0.005
*Yes*	7 (5.4%)	51 (1.9%)		4 (5.1%)	51 (1.1%)	
Chest pain			<0.001			<0.001
*Yes*	37 (28.7%)	406 (15.0%)		26 (32.9%)	523 (11.0%)	
Shortness of breath			<0.001			<0.001
*Yes*	28 (21.7%)	311 (11.5%)		20 (25.3%)	444 (9.3%)	
Ever diagnosed with DM			0.085			NA
*Yes*	3 (2.3%)	168 (6.2%)		0 (0%)	110 (2.3%)	
TB history			0.148			0.122
*Personal*	39 (30.2%)	864 (31.8%)		8 (10.1%)	880 (18.5%)	
*Household*	20 (15.5%)	275 (10.1%)		6 (7.6%)	283 (5.9%)	
W4SS			0.003			<0.001
*Yes*	76 (58.9%)	1230 (45.3%)		64 (81.0%)	1719 (36.1%)	

BMI – body mass index, DM – diabetes mellitus, NA – not available, SD – standard deviation, TB – tuberculosis, W4SS – WHO-recommended four-symptom screen, x̄ – mean

*P-values are calculated by *t*-test and Pearson χ^2^ test for continuous variables and categorical variables respectively. For those variables with 20% cells less than 5, Fisher exact test is applied to compute *P*-values.

**Table 2:** Characteristics of the HIV-negative/unknown population [11]

Variables	South Africa			Zambia		
	**TB prevalence = 2.0%**			**TB prevalence = 0.36%**		
	**TB**	**no TB**	*P*-value*	**TB**	**no TB**	*P*-value*
	**(n = 531)**	**(n = 26 343)**		**(n = 107)**	**(n = 29 275)**	
Sex			<0.001			0.001
*Male*	271 (51.0%)	10296 (39.1%)		54 (50.5%)	10327 (35.3%)	
Age, years			<0.001			0.274
*x̄ (SD)*	37.3 (15.2)	34.2 (14.0)		31.3 (12.9)	32.7 (14.8)	
*Missing*		18 (0.1%)			388 (1.3%)	
BMI, kg/m^2^			<0.001			<0.001
*x̄ (SD)*	23.3 (6.00)	27.5 (7.23)		21.0 (3.43)	22.9 (4.32)	
*Missing*	354 (66.7%)	17255 (65.5%)		11 (10.3%)	2897 (9.9%)	
Ever smoke			<0.001			<0.001
*Yes*	208 (39.2%)	6544 (24.8%)		32 (29.9%)	3738 (12.8%)	
Ever drink			<0.001			<0.001
*Yes*	322 (60.6%)	11851 (45.0%)		66 (61.7%)	12556 (42.9%)	
Productive cough			<0.001			<0.001
*Yes*	132 (24.9%)	2454 (9.3%)		31 (29.0%)	1964 (6.7%)	
Cough			<0.001			<0.001
*Cough> = 2 wks*	102 (19.2%)	1252 (4.8%)		17 (15.9%)	806 (2.8%)	
*Cough <2 wks*	63 (11.9%)	1840 (7.0%)		19 (17.8%)	2224 (7.6%)	
Fever			<0.001			<0.001
*Yes*	133 (25.0%)	4707 (17.9%)		14 (13.1%)	1389 (4.7%)	
Night sweats			<0.001			<0.001
*Yes*	157 (29.6%)	4011 (15.2%)		16 (15.0%)	1481 (5.1%)	
Weight loss			<0.001			0.124
*Yes*	146 (27.5%)	3433 (13.0%)		18 (16.8%)	3392 (11.6%)	
Haemoptysis			<0.001			<0.001
*Yes*	12 (2.3%)	202 (0.8%)		5 (4.7%)	111 (0.4%)	
Chest pain			<0.001			<0.001
*Yes*	94 (17.7%)	2544 (9.7%)		22 (20.6%)	2246 (7.7%)	
Shortness of breath			<0.001			<0.001
*Yes*	81 (15.3%)	2073 (7.9%)		18 (16.8%)	1539 (5.3%)	
Ever diagnosed with DM			0.947			0.132
*Yes*	40 (7.5%)	2030 (7.7%)		4 (3.7%)	532 (1.8%)	
TB history			<0.001			0.017
*Personal*	95 (17.9%)	2686 (10.2%)		9 (8.4%)	990 (3.4%)	
*Household*	86 (16.2%)	3564 (13.5%)		7 (6.5%)	1447 (4.9%)	
*Missing*		10 (0.0%)			1 (0.0%)	
Any TB symptom†			<0.001			<0.001
*Yes*	289 (54.4%)	9178 (34.8%)		44 (41.1%)	7027 (24.0%)	

BMI – body mass index, DM – Diabetes mellitus, SD – standard deviation, TB – tuberculosis, x̄ – mean

*P-values are calculated by *t*-test and Pearson χ^2^ test for continuous variables and categorical variables respectively. For those variables with 20% cells less than 5, Fisher exact test is applied to compute *P*-values.

†Cough, fever, night sweats, weight loss, haemoptysis.

### Prediction model development and validation

The predictors selected in the final model were different between the HIV-positive population and HIV-negative/unknown population.

#### HIV-positive population

**Figure 1:**
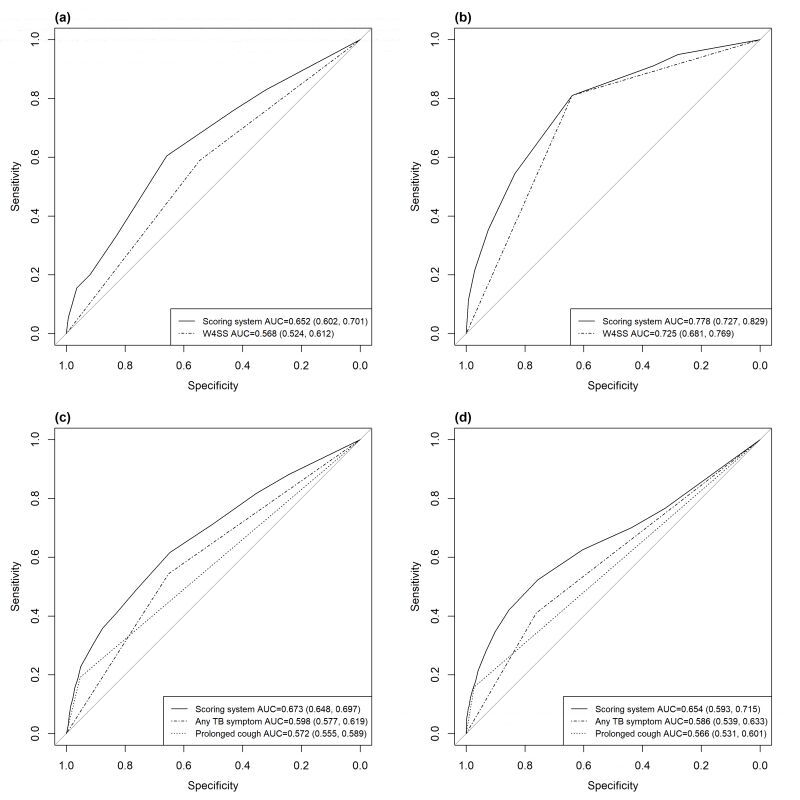
Receiver Operating Characteristic (ROC) curve of the scoring system among two populations from different countries. **Panel A.** ROC curve of the model for HIV-positive population applied in South African data set. **Panel B.** ROC curve of the model for HIV-positive population applied in Zambian data set (external validation). **Panel C.** ROC curve of the model for HIV-negative/unknown population applied in South African data set. **Panel D.** ROC curve of the model for HIV-negative/unknown population applied in Zambian data set (external validation).

Selected predictors and corresponding scores are shown in **Table 3**. The range for the total score was zero to seven. In the South African data set, the AUC of our scoring system was 0.652 (95% CI = 0.602–0.701), which was higher than that of W4SS (AUC = 0.568, 95% CI = 0.524–0.612, *P* < 0.001) (**Figure 1**, Panel A). In the external validation done on the Zambian data set, the AUC of the scoring system was 0.778 (95% CI = 0.727–0.829), which was still significantly different from that of W4SS (AUC = 0.725, 95% CI = 0.681–0.769, *P* = 0.025) (**Figure 1**, Panel B). Prolonged cough could not be compared here since chronic cough has been found insensitive for TB screening among HIV patients [7,21].

**Table 3:** Summary of the final model and the score corresponding to each predictor

Variables	Estimate	Score	Score (rounded)	OR (95% CI)
Model for HIV-positive population				
*Weight loss*	0.30	1.000	1	1.4 (0.9–2.0)
*Sex*	0.32	1.036	1	1.4 (0.9–2.0)
*Ever drink*	0.52	1.697	2	1.7 (1.1–2.5)
*Cough*	0.66	2.165	2	1.9 (1.3–2.9)
*Chest pain*	0.39	1.283	1	1.5 (0.9–2.3)
Model for HIV-negative/unknown population				
*Weight loss*	0.46	2.640	3	1.6 (1.3–2.0)
*Night sweats*	0.29	1.658	2	1.3 (1.1–1.6)
*Ever smoke*	0.17	1.000	1	1.2 (1.0–1.5)
*Sex*	0.24	1.381	1	1.3 (1.0–1.5)
*Ever drink*	0.32	1.836	2	1.4 (1.1–1.7)
*Cough <2 weeks*	0.56	3.246	3	1.8 (1.3–2.3)
*Cough ≥2 weeks*	1.22	7.025	7	3.4 (2.6–4.3)
*Personal TB history*	0.23	1.324	1	1.3 (1.0–1.6)
*Household TB history*	0.33	1.900	2	1.4 (1.1–1.8)

CI – confidence interval, OR – odds ratio, TB – tuberculosis

#### HIV-negative/unknown population

Selected predictors and corresponding scores are shown in **Table 3**. The range for the total score was zero to 18. In the South African data set, the AUC of our scoring system was 0.673 (95% CI = 0.648–0.697), which was higher than that of any TB symptom (AUC = 0.598, 95% CI = 0.577–0.619, *P* < 0.001) and that of prolonged cough (AUC = 0.572, 95% CI = 0.555–0.589, *P* < 0.001) (**Figure 1**, Panel C). In the Zambian data set, the AUC of our scoring system was 0.654 (95% CI = 0.593–0.715), which was also higher than the performance of WHO tools. *P* for the difference compared with any TB symptom or prolonged cough was 0.001 and 0.002 respectively (**Figure 1**, Panel D).

### Internal validation

For internal validation, the distribution of the AUC of the models developed among 1000 bootstrapped samples is shown in Figure S4 in the **Online Supplementary Document**. Among the HIV-positive population, the mean of 1000 AUC generated from bootstrapped scoring systems was 0.668 (95% CI = 0.616–0.720), which was similar to that of our scoring system (AUC 0.652). A similar result was shown among the HIV-negative/unknown population.

### Application of the scoring system

In the field, the scoring system for both the HIV-positive and HIV-negative/unknown populations could be transformed into a practical TB screening score sheet as shown in Text S2 in the **Online Supplementary Document**. Public health care workers could choose an appropriate scoring system according to the HIV status of individuals, and the screening sheet will assist in triaging individuals in the community. Table S3 in the **Online Supplementary Document** shows the sensitivity and specificity at different cut-off points of the total scores.

### CEA results of possible active TB case finding strategies

**Figure 2:**
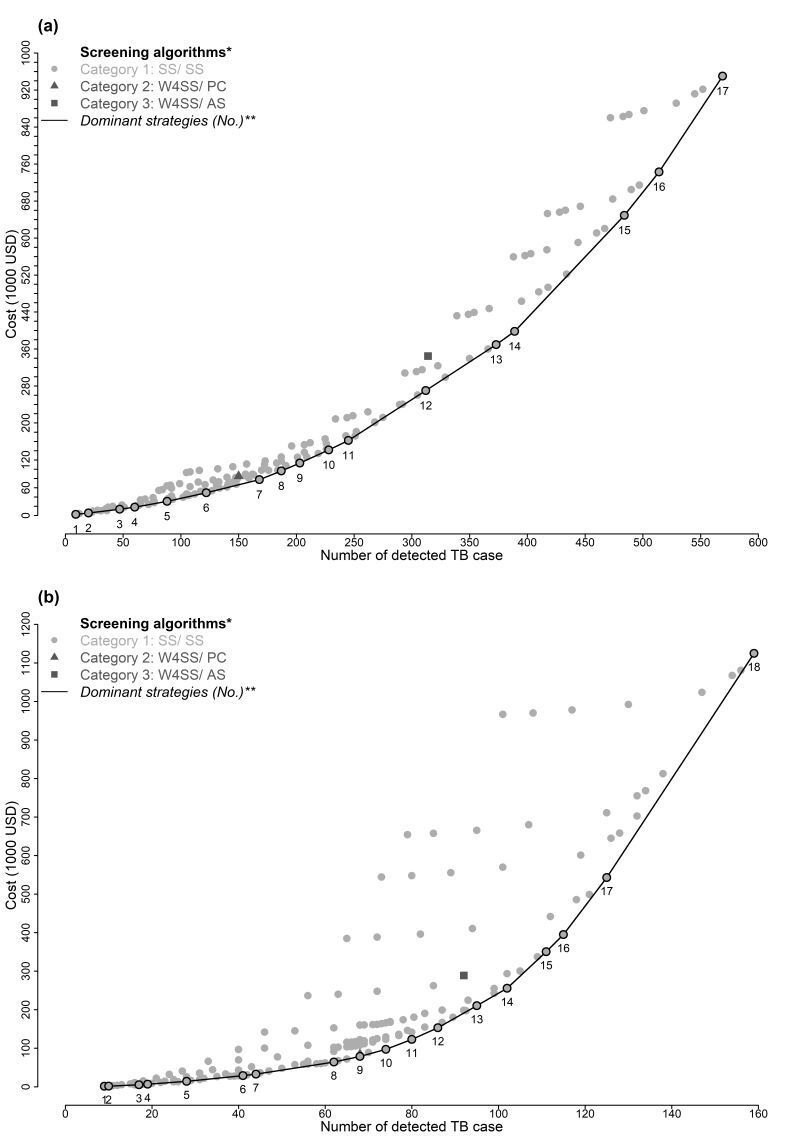
Cost-effectiveness plane. **Panel A.** The result in South African data set. **Panel B.** The result in Zambian data set. Current figure shows the result of three categories of screening algorithms explained in Table S1 in the **Online Supplementary Document****.** Details of selected dominant strategies are shown in **Table 4** and **Table 5**. *Left of slash: the screening algorithms for HIV-positive populations; right of slash: the screening algorithms for HIV-negative/unknown populations. AS – any tuberculosis symptom, PC – prolonged cough, SS – scoring system, W4SS – WHO-recommended four-symptom screen.

**Figure 2**, Panels A–B shows the cost-effectiveness plane of our TB screening tool applied to the South African and the Zambian data sets, by applying the strategies mentioned in Table S1 in the **Online Supplementary Document**. On the cost-effectiveness plane of the South African data set, the tools proposed by WHO and most of the cut-off point groups were dominated by 17 cut-off point groups. Similar results were found in the Zambian data set. **Table 4** and **Table 5** show the detailed information on these strategies. The ACER ranged from 246 to 1670 USD in the South African data set, and from 164 to 7074 USD in the Zambian data set, when these strategies were applied.

**Table 4:** Detail of the screening strategies which dominated other competing algorithms (dots on the curve on cost-effectiveness plane) in the South African data set*

No.	Cut-off points of the scoring system		Number of the detected TB cases (95% CI)	Proportion of the detected TB cases (95% CI)	Cost in USD (95% CI)	ACER (95% CI)	ICER
	**HIV-positive**	**HIV-negative/unknown**					
1	7	18	9 (4–15)	1.4% (0.6–2.3%)	2272 (1760–2816)	246 (162–512)	252
2	6	18	20 (13–27)	3.0% (2.0–4.1%)	5248 (4479–6017)	267 (194–406)	271
3	6	15	47 (34–59)	7.1% (5.2–8.9%)	13 440 (12256–14624)	289 (230–385)	303
4	6	14	60 (46–73)	9.1% (7.0–11.1%)	17 888 (16512–19168)	301 (245–381)	342
5	6	12	88 (71–106)	13.3% (10.8–16.1%)	30 560 (28608–32321)	348 (294–426)	453
6	6	10	122 (103–142)	18.5% (15.6–21.5%)	49 056 (46624–51329)	402 (346–473)	544
7	3	10	168 (147–191)	25.5% (22.3–28.9%)	77 408 (74784–80162)	462 (406–527)	616
8	3	9	187 (164–211)	28.3% (24.8–32.0%)	96 224 (93408–99330)	516 (455–586)	990
9	3	8	203 (179–230)	30.8% (27.1–34.8%)	113 344 (110144–116800)	558 (495–634)	1070
10	3	7	228 (204–258)	34.5% (30.9–39.1%)	141 696 (137984–145344)	620 (550–699)	1134
11	2	7	245 (217–274)	37.1% (32.9–41.5%)	162 208 (158559–165890)	665 (595–744)	1207
12	1	5	312 (283–345)	47.3% (42.9–52.3%)	269 936 (265086–274401)	865 (780–956)	1608
13	1	4	373 (338–409)	56.5% (51.2–62.0%)	369 504 (364064–374753)	991 (902–1093)	1632
14	0	4	389 (355–427)	58.9% (53.8–64.7%)	398 112 (393310–403010)	1023 (930–1119)	1788
15	0	2	484 (446–525)	73.3% (67.6–79.6%)	649 248 (644160–653825)	1342 (1237–1452)	2644
16	0	1	514 (475–557)	77.9% (72.0–84.4%)	743 104 (738686–747169)	1448 (1335–1565)	3129
17	0	0	569 (528–613)	86.2% (80.0–92.9%)	950 048 (950048–950048)	1670 (1550–1799)	3763
▲	W4SS	PC	150 (129–172)	22.7% (19.5–26.1%)	85 056 (82336–87809)	569 (494–657)	X
■	W4SS	AS	314 (283–349)	47.6% (42.9–52.9%)	344 576 (339263–349601)	1096 (988–1214)	X

ACER – average cost-effectiveness ratio, AS – any TB symptom, ICER – incremental cost-effectiveness ratio, PC – prolonged cough, TB – tuberculosis, W4SS – WHO-recommended four-symptom screen

*The result of the cost-effectiveness analysis in the South African data set, which included 17 cut-off point groups, are presented. The last two strategies listed in the table (▲: W4SS-PC and ■: W4SS-AS) were dominated by the aforementioned 17 cut-off groups.

**Table 5:** Detail of the screening strategies which dominated other competing algorithms (dots on the curve on cost-effectiveness plane) in the Zambian data set*

No.	Cut-off points of the scoring system			Number of the detected TB cases (95% CI)		Proportion of the detected TB cases (95% CI)		Cost (USD) (95% CI)		ACER (95% CI)		ICER	
	**HIV-positive**	**HIV-negative/unknown**											
1	7		18		9 (4–15)		4.8% (2.2–8.1%)		1512 (1100–1978)		164 (99–376)		168
2	7		17		10 (5–16)		5.4% (2.7–8.6%)		1812 (1344–2294)		181 (108–362)		300
3	6		17		17 (10–25)		9.1% (5.4–13.4%)		5266 (4520–6084)		314 (215–538)		493
4	6		15		19 (12–28)		10.2% (6.5–15.1%)		6829 (5956–7756)		354 (247–570)		782
5	5		16		28 (19–38)		15.1% (10.2–20.4%)		13 995 (12 802–15 304)		508 (369–749)		796
6	4		15		41 (30–53)		22.0% (16.1–28.5%)		28 906 (27 288–30 687)		702 (544–958)		1147
7	4		13		44 (32–56)		23.7% (17.2–30.1%)		32 883 (31 010–34 754)		747 (586–1010)		1326
8	3		13		62 (48–76)		33.3% (25.8–40.9%)		64 151 (61 861–66 470)		1042 (843–1339)		1737
9	3		10		68 (54–82)		36.6% (29.0–44.1%)		78 867 (76 398–81 758)		1163 (957–1480)		2453
10	3		8		74 (60–89)		39.8% (32.3–47.8%)		97 267 (94 456–100 554)		1324 (1096–1633)		3067
11	3		7		80 (65–96)		43.0% (34.9–51.6%)		122 884 (119 590–126 662)		1540 (1285–1906)		4270
12	3		6		86 (70–103)		46.2% (37.6–55.4%)		153 320 (149 640–157 437)		1786 (1480–2188)		5073
13	1		6		95 (79–113)		51.1% (42.5–60.8%)		210 544 (206 802–214 564)		2211 (1855–2684)		6358
14	1		5		102 (85–121)		54.8% (45.7–65.1%)		255 511 (251 457–259 910)		2510 (2113–3002)		6424
15	1		4		111 (94–130)		59.7% (50.5–69.9%)		350 326 (345 168–355 566)		3144 (2694–3731)		10535
16	0		4		115 (97–134)		61.8% (52.2–72.1%)		394 809 (389 863–399 411)		3435 (2941–4043)		11121
17	0		3		125 (106–145)		67.2% (57.0–78.0%)		543 021 (537 638–549 116)		4353 (3734–5115)		14821
18	0		0		159 (137–182)		85.5% (73.6–97.9%)		1 124 796 (1 124 316–1 125 308)		7074 (6177–8215)		17111
▲	W4SS		PC		68 (54–83)		36.6% (29.0–44.6%)		84 180 (81 280–87 033)		1231 (1012–1552)		X
■	W4SS		AS		92 (75–110)		49.5% (40.3–59.1%)		288 636 (283 609–293 990)		3140 (2629–3817)		X

ACER – average cost-effectiveness ratio, AS – any TB symptom, ICER – incremental cost-effectiveness ratio, PC – prolonged cough, TB – tuberculosis, W4SS – WHO-recommended four-symptom screen

*The result of the cost-effectiveness analysis in the Zambian data set, which included 18 cut-off point groups, are presented. The last two strategies listed in the table (▲: W4SS-PC and ■: W4SS-AS) were dominated by the aforementioned 18 cut-off groups.

As for the result of extended application of CEA, Figure S5 in the **Online Supplementary Document** showed the ACER of the 17 strategies selected in the South African data set in 16 situations. The difference between ACER could only be significantly distinguished when the prevalence of TB was different. Figure S6 in the **Online Supplementary Document** showed that 15 cut-off point groups were selected for active TB case finding in the application of Zimbabwean data. Above indicated that the algorithms proposed by WHO were still dominated by some cut-point groups even when we inputted real-world data to the cost-effectiveness analysis.

## DISCUSSION

A prediction model for active TB case finding was developed and validated in our study. The model was separated into two scoring systems, for a HIV-positive population and a HIV-negative/unknown population, respectively. The selected predictors with assigned scores could be integrated into a score sheet for active TB case finding in communities. Moreover, 17 and 18 cut-off point groups that dominated other competing strategies among the South African and Zambian data sets. The ACER therefore ranged from 246 to 1670 USD and from 164 to 7074 USD among two study populations.

In the HIV-positive population, the prediction model had a significantly better performance than the W4SS rule in both the South African and Zambian data sets. However, the effect of different stages of HIV infection, taking antiretroviral therapy (ART), and CD4 T lymphocyte counts were not considered candidate predictors in our model due to under measurement and a large proportion of missing data. This made the content of our model different among existing algorithms for active TB cases living with HIV [22–24].

As for the HIV-negative/unknown population, the prediction model’s performance was significantly better than WHO tools. The AUC of the prediction model seemed not to differ between the two countries, which indicated a stable discrimination ability of our model for HIV-negative/unknown populations. Nonetheless, since some participants did not receive HIV tests and self-reported HIV status was used instead [14], the observed effect of selected predictors may be affected and therefore made the prediction inaccurate. However, that might be a trade-off because revealing the HIV status of all residents in a community would bring extra financial burden and the social issue of stigma.

The result of extended application showed that ACER would be affected mainly by TB prevalence (Figure S5 in the **Online Supplementary Document**). Through a combination of the prediction model and cost-effectiveness analysis, the tool developed in our study could help TB programmes do active TB case finding implementation in communities, assisting the decision on the ideal screening strategy while considering a limited budget and effectiveness.

In this study, we developed and validated a prediction model for active TB case finding in a community setting and made the model simple and feasible by converting the logistic regression model into a scoring system. We considered the nature of HIV-positive and HIV-negative/unknown populations, and built two separate prediction models. The predictors in the model could be measured conveniently by trained clinical staff rather than performing laboratory tests. Furthermore, we compared the screening strategies for TB including the tools proposed by WHO with our scoring systems based on the number of TB cases detected and the corresponding cost. We showed that our scoring system could perform better and be more efficient than the symptom-based algorithms that WHO recommends. In addition, several cut-off point groups within the scoring system could provide various options for TB screening under a constrained budget.

Our study still has some limitations. First, the effect of selected predictors on active TB may be confounded. The model could not reflect the effect of stage and treatment of HIV, and HIV-negative and unknown populations share only one model, which may limit the generalisability of the scoring systems developed in our study. Second, we only considered the cost of diagnostic tools, which might lead to an underestimation of ACER. It would be more complete to add all types of costs generated in community-based screening programmes for a short-term evaluation [25]. Third, we could not estimate ACF's effectiveness since we did not aim to make a comparison with PCF at baseline. ACF may even have some impact on TB transmission, which we did not account for. Finally, we obtained data from Southern African countries only. The predictors selected into the models might differ in other countries or regions due to diverse epidemiological characteristics.

Therefore, in this study, we could only provide an approach that demonstrates a trade-off between budget considerations and infectious disease control strategies, to assist in decision making. Our evidence shows that our scoring system could dominate the symptom-based TB screening strategies proposed by the WHO for resource-constrained settings. Future research could build on this study by conducting economic evaluations using Disability-Adjusted Life Years or Quality-Adjusted Life Years to gain valuable insights into intervention impacts on health and inform evidence-based health care decision-making.

## CONCLUSIONS

A prediction model for active TB case finding was developed and presented in the form of a scoring system, which would be feasible and practical for use in community-based active TB case finding programmes. Several cut-off point groups selected in both the South African and Zambian data sets could identify the largest number of TB cases detected while considering different programmatic budget constraints. The ACER in the two data sets indicated the high cost of finding a TB case. However, this study demonstrates an approach and provides a tool for TB programmes to actively find missing TB cases, which would be useful information for future research.

## Additional material


Online Supplementary Document
