# Supplementary material for: Cost-effectiveness analysis of a prediction model for community-based screening of active tuberculosis
Source: J Glob Health. 2024 Nov 22;14:04226. doi: 10.7189/jogh.14.04226 (PMC11586587; doi:10.7189/jogh.14.04226)
Supplement: Online Supplementary Document [file jogh-14-04226-s001.pdf]

Supporting information

Figure S1. Flow of the study methods.

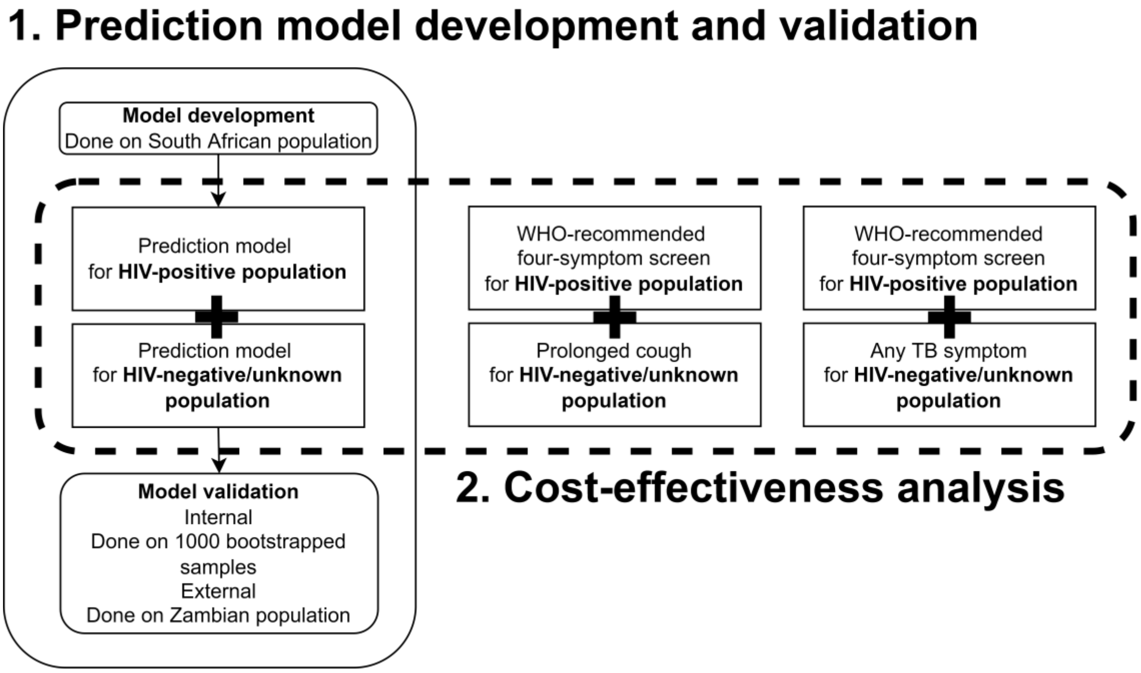

Figure S2. Flow chart of the study and sample size.

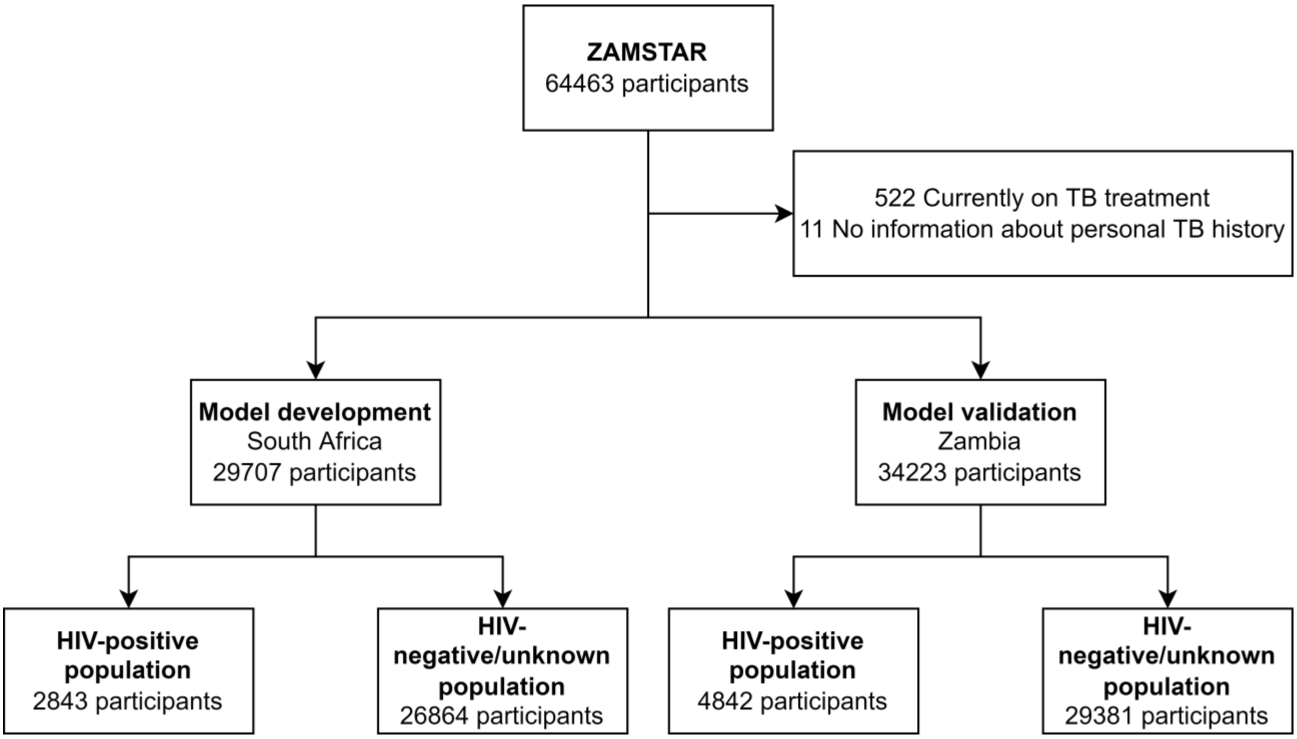

Figure S3. Flow chart of the clinical algorithm.

The flow chart shows the possible process from screening to diagnosis.

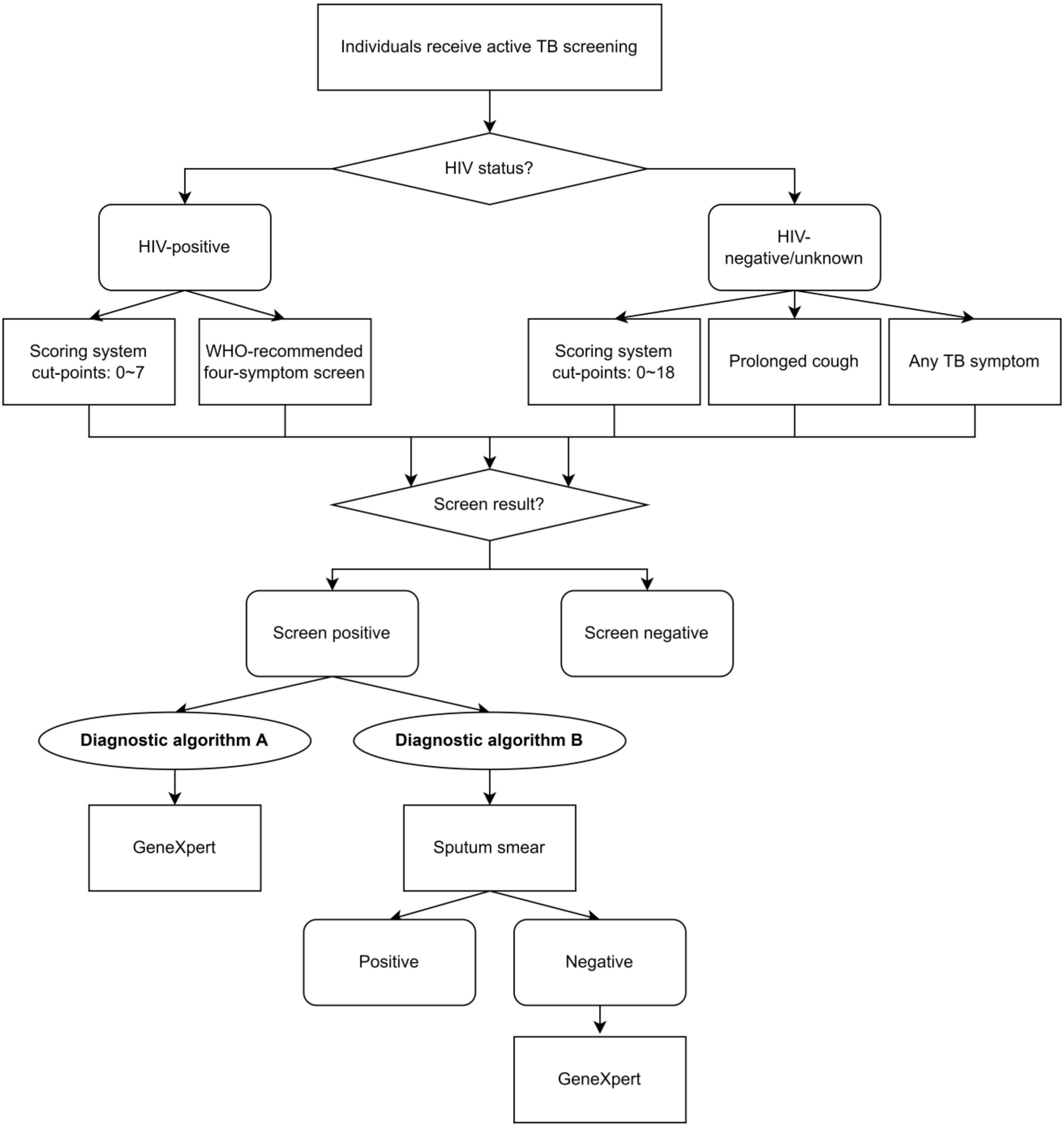

Figure S4. The histogram of the distribution of the AUC of 1000 models developed on the bootstrapped samples.

(a). Among HIV-positive population

(b). Among HIV-negative/unknown population

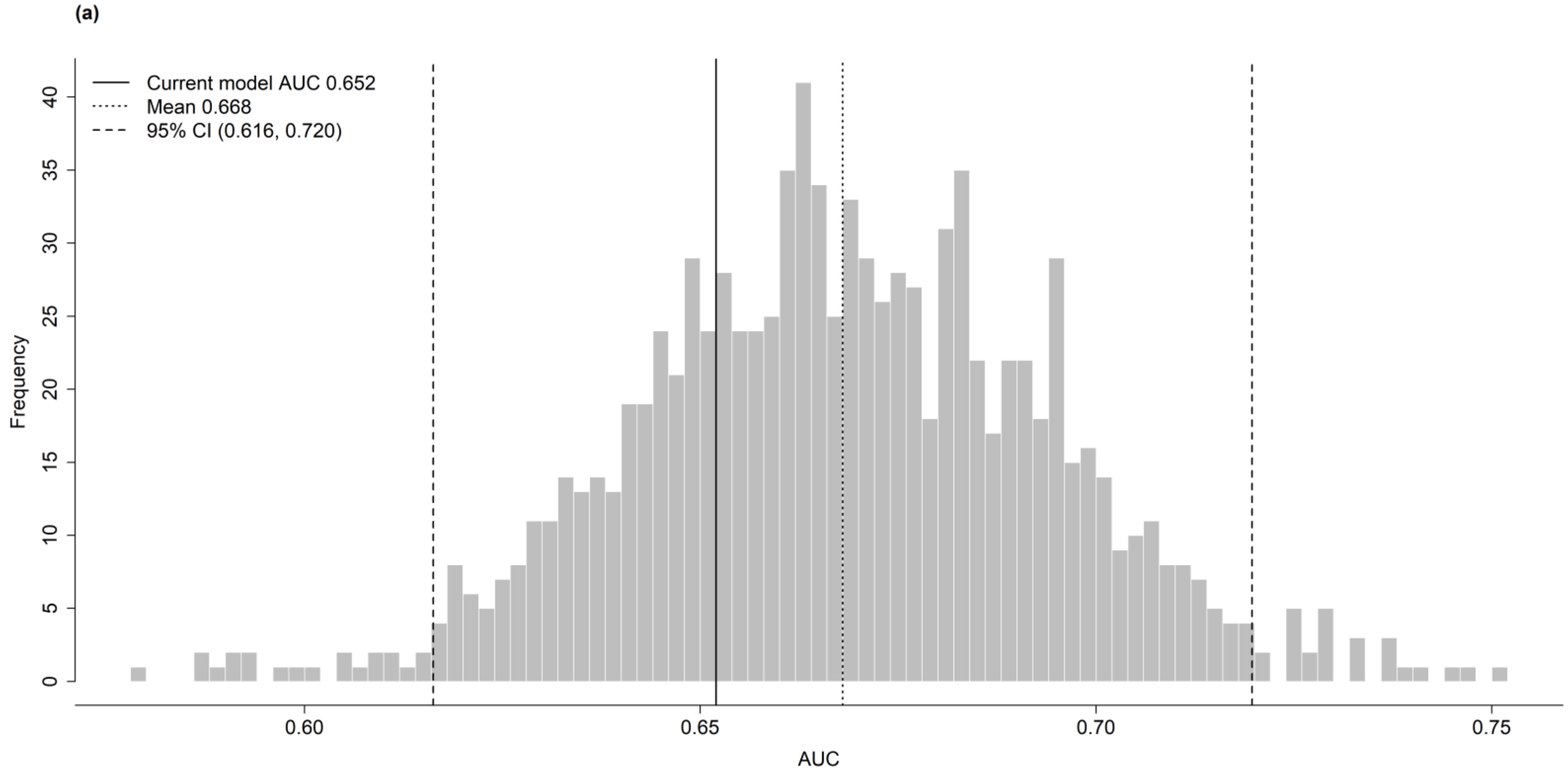

(b)

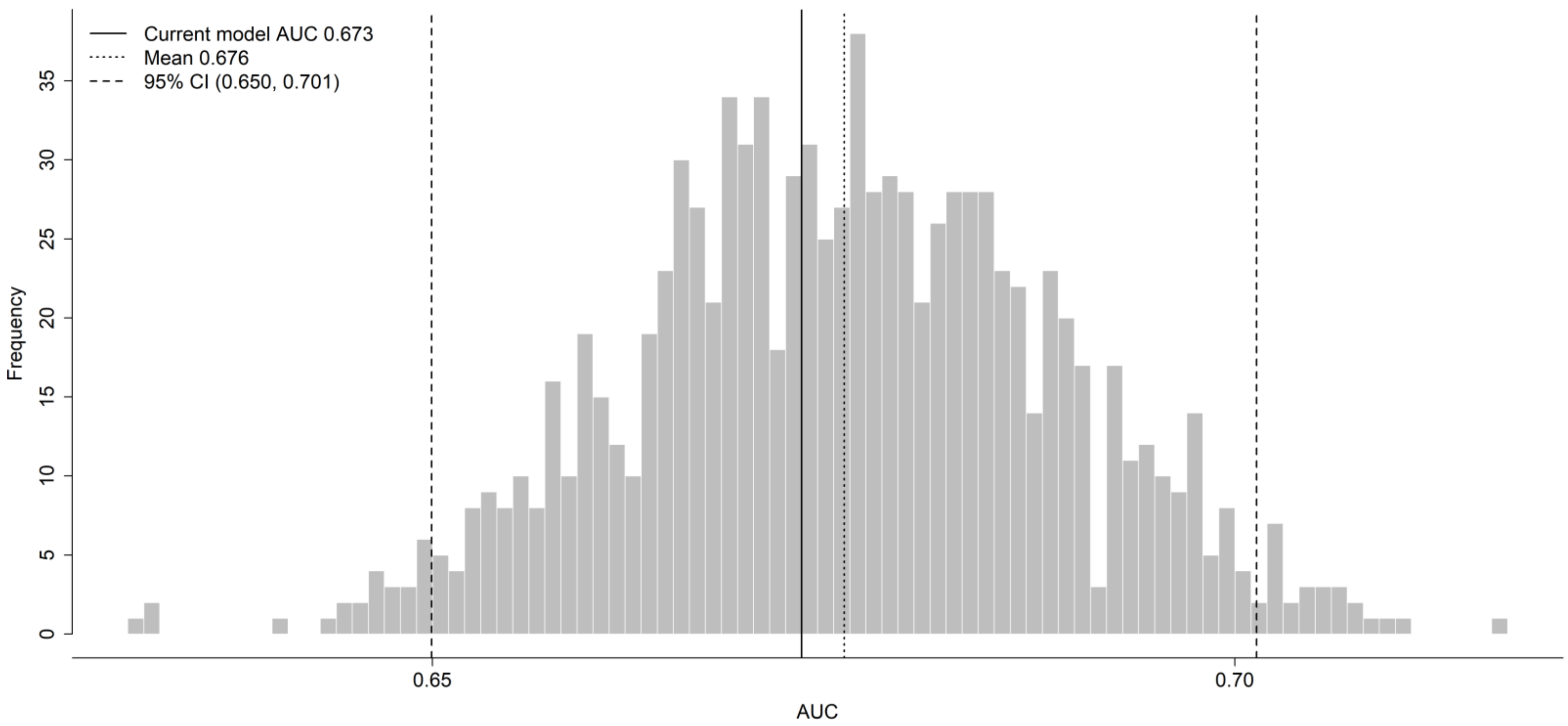
Current model AUC 0.673
Mean 0.676
95% CI (0.650, 0.701)
Frequency
0
5
10
15
20
25
30
35
0.65
0.70
AUC

Figure S5. Application of the CEA results using hypothetical populations with different epidemiological characteristics.

Several hypothetical populations in different proportion of TB population living with HIV by using the strategies selected in South African dataset. (a). 10% of TB population living with HIV; (b). 30% of TB population living with HIV; (c). 50% of TB population living with HIV; (d). 70% of TB population living with HIV.

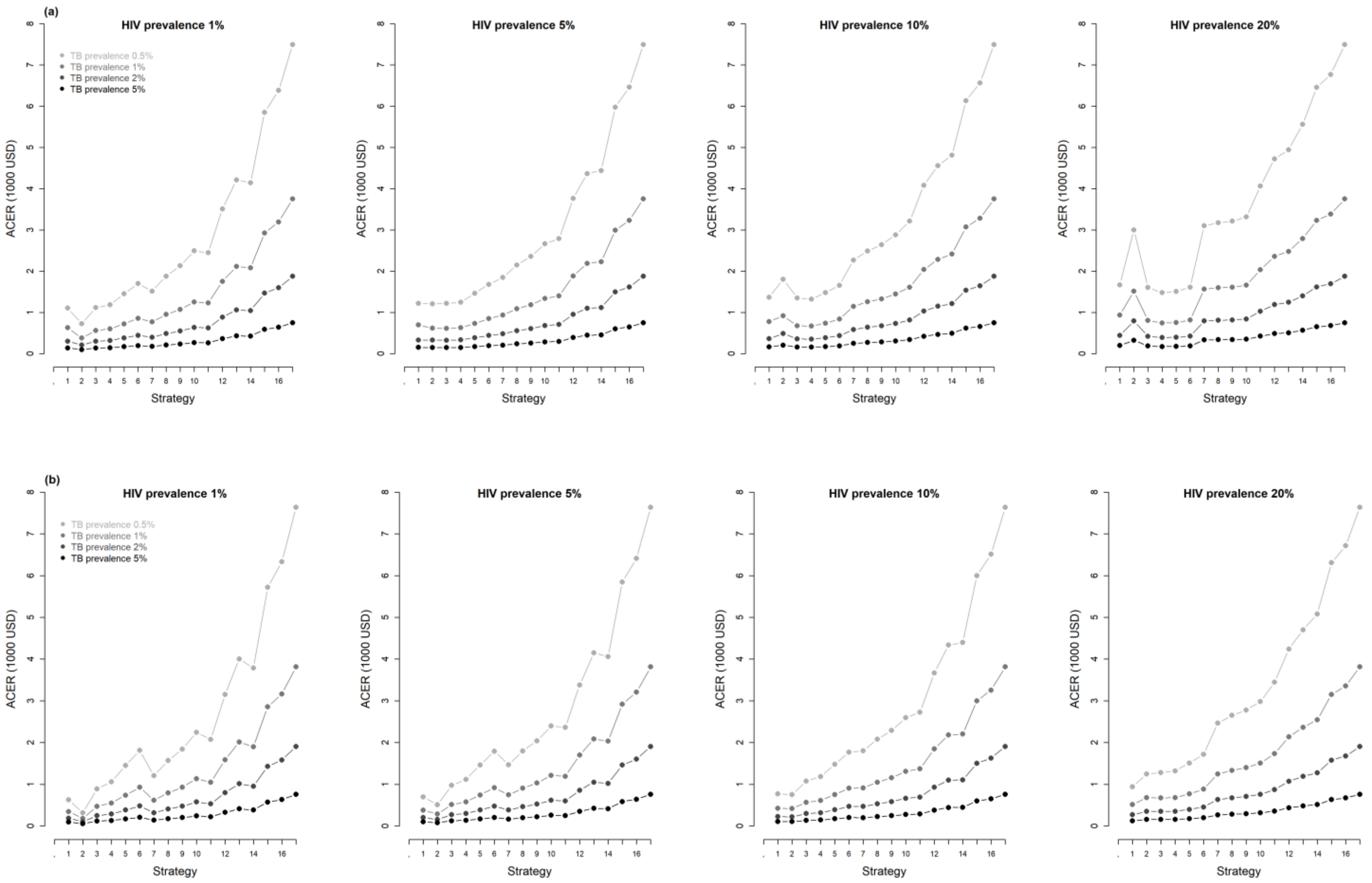

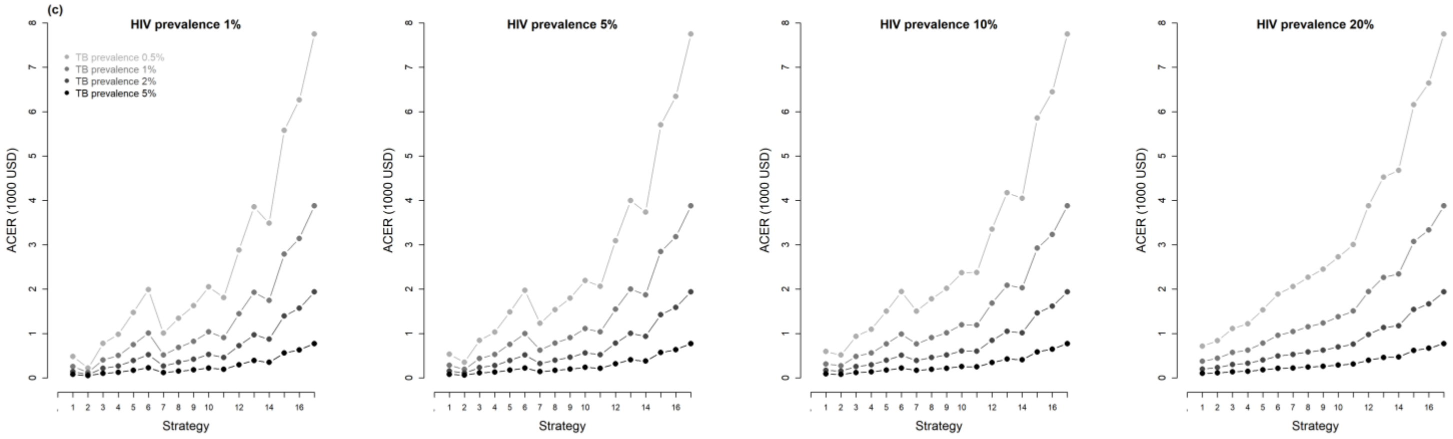
(c)
HIV prevalence 1%
HIV prevalence 5%
HIV prevalence 10%
HIV prevalence 20%
TB prevalence 0.5%
TB prevalence 1%
TB prevalence 2%
TB prevalence 5%
ACER (1000 USD)
Strategy

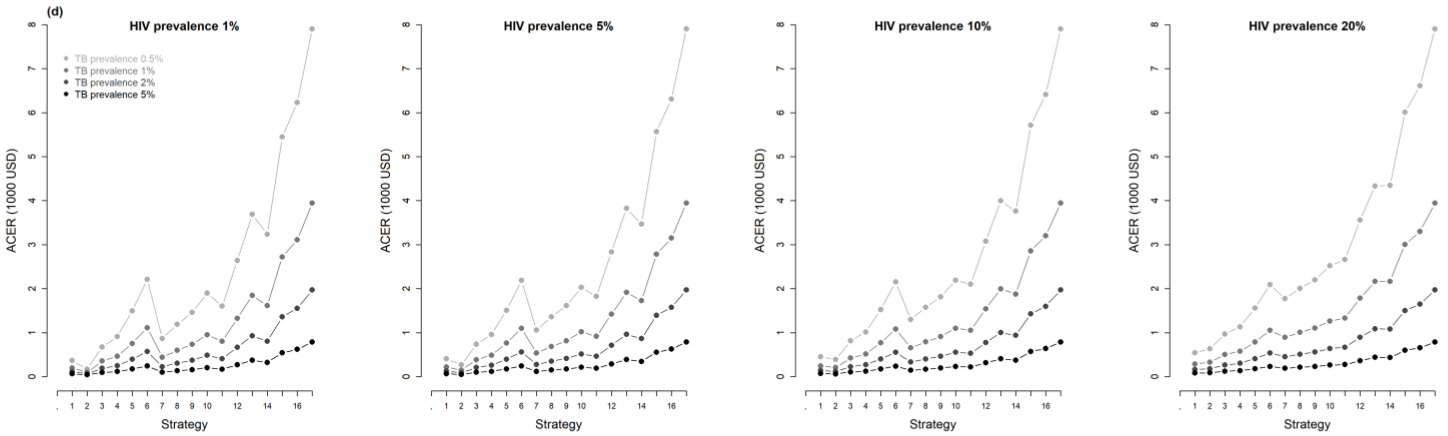
(d)
HIV prevalence 1%
HIV prevalence 5%
HIV prevalence 10%
HIV prevalence 20%
TB prevalence 0.5%
TB prevalence 1%
TB prevalence 2%
TB prevalence 5%
ACER (1000 USD)
Strategy

Figure S6. Cost-effectiveness plane of the community in Zimbabwe.

The data of HIV prevalence, TB prevalence and the proportion of TB patients living with HIV were extracted from Corbett et al., 2010.

Current figure shows the result of 3 categories of screening algorithms explained in Table 1; *Left of slash: The screening algorithms for HIV-positive populations; Right of slash: The screening algorithms for HIV-negative/unknown populations; **SS:** scoring system; **W4SS:** WHO-recommended four-symptom screen; **AS:** any TB symptom; **PC:** prolonged cough

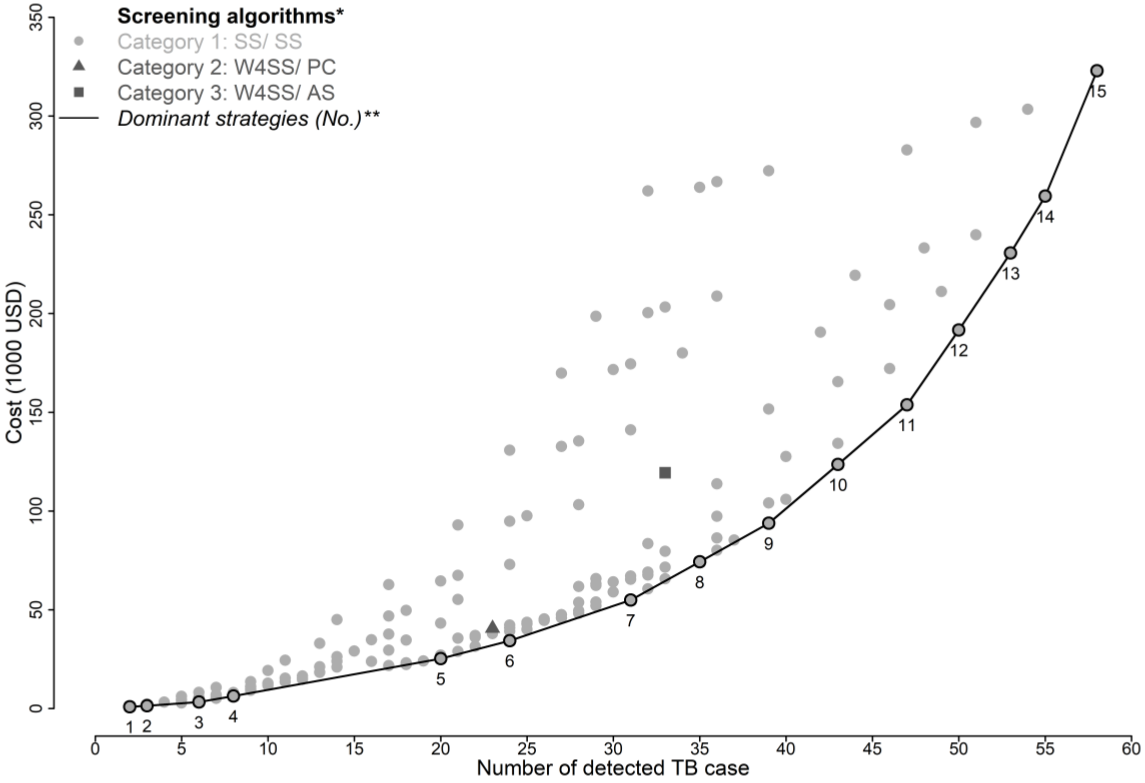

No.	Cut-off points HIV-positive	Cut-off points HIV-negative/ unknown
**1**	7	18
**2**	7	17
**3**	6	17
**4**	6	14
**5**	3	14
**6**	3	10
**7**	1	10
**8**	1	7
**9**	0	7
**10**	0	5
**11**	0	4
**12**	0	3
**13**	0	2
**14**	0	1
**15**	0	0

(HIV prevalence 19%; TB prevalence 0.7%; proportion of TB patients living with HIV 51%; population size: 10092)

Table S1. The possible active tuberculosis screening algorithms for HIV-positive and HIV-negative/unknown populations.

Type	Category	HIV-positive populations	HIV-negative/unknown populations	Note
Prediction model	1	Scoring system (cut-points: 0~7)	Scoring system (cut-points: 0~18)	Include 152 (8*19) cut-points groups
Symptom screening tools proposed by WHO [1]	2	WHO-recommended four-symptom screen (W4SS)*	Prolonged cough	X
	3	WHO-recommended four-symptom screen (W4SS)	Any TB Symptom**	X
		Total number of screening strategies:		154

*Cough, fever, night sweats, weight loss

**Cough, fever, night sweats, weight loss, haemoptysis

1. World Health Organization. WHO consolidated guidelines on tuberculosis Module 2: Screening – Systematic screening for tuberculosis disease. Geneva: World Health Organization,; 2021.

Table S2. The cost and the validity of the tools applied in the diagnostic algorithm.

		HIV-positive		HIV-negative/unknown	
		Sensitivity	Specificity	Sensitivity	Specificity
Diagnostic algorithm A	GeneXpert 32 USD[2]	79%[1] (70%-86%)	98%[1] (96%-99%)	86%[1] (76%-92%)	99%[1] (98%-100%)
Diagnostic algorithm B	Sputum smear 2 USD[4]	61%[3] (31%-89%)	98%[3] (93%-100%)	61%[3] (31%-89%)	98%[3] (93%-100%)
	GeneXpert 32 USD	61%[1] (40%-81%)	99%[1] (97%-100%)	67%[1] (60%-74%)	99%[1] (98%-99%)

Reference

1. Steingart, K.R., et al., *Xpert® MTB/RIF assay for pulmonary tuberculosis and rifampicin resistance in adults.* Cochrane Database of Systematic Reviews, 2014(1).
2. Meyer-Rath, G., et al., *The Impact and Cost of Scaling up GeneXpert MTB/RIF in South Africa.* PLOS ONE, 2012. **7**(5): p. e36966.
3. World Health Organization. *Systematic Screening for Active Tuberculosis: Principles and Recommendations*. Geneva: World Health Organization,; 2013.
4. Lu, C., et al., *A systematic review of reported cost for smear and culture tests during multidrug-resistant tuberculosis treatment.* PLoS One, 2013. **8**(2): p. e56074.

Table S3. The sensitivity and specificity at different cut-off points.

(a) was the scoring system for HIV-positive populations, (b) was the scoring system for HIV-negative/unknown populations. All values were shown as the median and 95% CI of the results among 1000 bootstrapped samples.

(a)

Cut-off points	Sensitivity (95% CI)	Specificity (95% CI)
7	0.054 (0.020, 0.097)	0.994 (0.990, 0.996)
6	0.157 (0.100, 0.221)	0.965 (0.958, 0.971)
5	0.203 (0.136, 0.272)	0.919 (0.908, 0.929)
4	0.336 (0.256, 0.411)	0.829 (0.814, 0.844)
3	0.606 (0.530, 0.692)	0.659 (0.641, 0.676)
2	0.761 (0.684, 0.835)	0.431 (0.412, 0.451)
1	0.831 (0.759, 0.897)	0.322 (0.303, 0.339)
0	1.000 (1.000, 1.000)	0.000 (0.000, 0.000)
W4SS	0.590 (0.507, 0.674)	0.547 (0.528, 0.566)

(b)

Cut-off points	Sensitivity (95% CI)	Specificity (95% CI)
18	0.007 (0.002, 0.015)	0.998 (0.998, 0.999)
17	0.014 (0.006, 0.026)	0.997 (0.996, 0.997)
16	0.031 (0.018, 0.047)	0.993 (0.992, 0.994)
15	0.065 (0.046, 0.086)	0.990 (0.989, 0.991)
14	0.093 (0.070, 0.118)	0.985 (0.984, 0.986)
13	0.118 (0.092, 0.145)	0.979 (0.977, 0.980)
12	0.154 (0.123, 0.184)	0.971 (0.969, 0.973)
11	0.184 (0.153, 0.217)	0.962 (0.959, 0.964)
10	0.227 (0.194, 0.262)	0.951 (0.948, 0.953)
9	0.267 (0.233, 0.304)	0.929 (0.926, 0.932)
8	0.303 (0.266, 0.342)	0.910 (0.906, 0.913)
7	0.358 (0.318, 0.399)	0.877 (0.873, 0.881)
6	0.408 (0.369, 0.451)	0.831 (0.826, 0.836)
5	0.488 (0.445, 0.530)	0.763 (0.758, 0.769)
4	0.616 (0.574, 0.660)	0.648 (0.642, 0.653)
3	0.713 (0.672, 0.749)	0.503 (0.497, 0.509)
2	0.817 (0.783, 0.852)	0.354 (0.348, 0.359)
1	0.882 (0.854, 0.908)	0.243 (0.239, 0.249)
0	1.000 (1.000, 1.000)	0.000 (0.000, 0.000)
Prolonged cough	0.192 (0.161, 0.225)	0.952 (0.950, 0.955)
Any TB symptom	0.545 (0.502, 0.587)	0.652 (0.646, 0.657)

Text S1. The process of the cost-effectiveness analysis.

Algorithm and the calculation of cost and effectiveness

The symptom-based screening algorithm recommended by WHO has two combinations:

i. W4SS for HIV-positive populations and prolonged cough for HIV-negative/unknown populations

ii. W4SS for HIV-positive populations and any TB symptom for HIV-negative/unknown populations

The screening algorithm is followed by two diagnostic algorithms shown in S2 Figure, diagnostic algorithm A and diagnostic algorithm B. Two different algorithms were designed under the consideration of the TB epidemics in South Africa and Zambia. Therefore, we assumed that the South African population applied the diagnostic algorithm A, and the Zambian population applied the diagnostic algorithm B. According to the S1 Table, we calculated the number of TB cases found through an algorithm and the total cost of the diagnostic tool needed to spend. We replicated the analyses under 1000 bootstrapped samples, and we computed the median number of detected TB cases and costs for plotting the cost-effectiveness plane.

Hypothetical analysis and extended application

In hypothetical analysis, we inputted different HIV prevalence, TB prevalence, and TB-HIV co-infection, aiming to determine how these three factors would affect the cost for TB case detection at different cut-off point groups. We applied the result of selected cut-off point groups in the South African population to perform the analysis. We observed the trend of ACER on different strategies. As for the extended application, we applied diagnostic algorithm A to generate the simulated number of detected TB cases and costs by extracting the data from a Zimbabwean study.

Text S2. The active tuberculosis screening sheet.

The screening sheet was designed according to the scoring systems converted by the prediction models.

Health workers can simply do active tuberculosis screening in the communities through this sheet. The sheet could be paper-based or modified to an e-form

Active tuberculosis screening sheet

- Ask the HIV status first. For HIV-positive individual, please use the table below; for HIV-negative/unknown individual, please use the table right side.
- Check the corresponding answer for each item and sum the total assigned score.

HIV-positive individual		Check	Associated points	Assigned score
**Gender**	Male	☐	1	
	Female	☐	0	
**Weight loss**	Yes	☐	1	
	No	☐	0	
**Current cough**	Yes	☐	2	
	No	☐	0	
**Ever drink**	Yes	☐	2	
	No	☐	0	
**Chest pain**	Yes	☐	1	
	No	☐	0	
		**TOTAL SCORE**		0~7

HIV-negative/unknown individual		Check	Associated points	Assigned score
**Gender**	Male	☐	1	
	Female	☐	0	
**Weight loss**	Yes	☐	3	
	No	☐	0	
**Cough**	Yes, >=2 weeks	☐	7	
	Yes, < 2 weeks	☐	3	
	No	☐	0	
**Ever drink**	Yes	☐	2	
	No	☐	0	
**Ever smoke**	Yes	☐	1	
	No	☐	0	
**Night sweats**	Yes	☐	2	
	No	☐	0	
**TB history**	Personal	☐	2	
	Household	☐	1	
	No	☐	0	
		**TOTAL SCORE**		0~18
